# Comparison of Natural Language Processing and Manual Coding for the Identification of Cross-Sectional Imaging Reports Suspicious for Lung Cancer

**DOI:** 10.1200/CCI.17.00069

**Published:** 2018-02-20

**Authors:** Roxanne Wadia, Kathleen Akgun, Cynthia Brandt, Brenda T. Fenton, Woody Levin, Andrew H. Marple, Vijay Garla, Michal G. Rose, Tamar Taddei, Caroline Taylor

**Affiliations:** **Roxanne Wadia**, **Kathleen Akgun**, **Cynthia Brandt**, **Brenda T. Fenton**, **Andrew H. Marple**, **Vijay Garla**, **Michal G. Rose**, and **Tamar Taddei**, Yale University School of Medicine, New Haven; and **Roxanne Wadia**, **Kathleen Akgun**, **Cynthia Brandt**, **Brenda T. Fenton**, **Woody Levin**, **Michal G. Rose**, **Tamar Taddei**, and **Caroline Taylor**, Veterans Affairs Connecticut Healthcare System, West Haven, CT.

## Abstract

**Purpose:**

To compare the accuracy and reliability of a natural language processing (NLP) algorithm with manual coding by radiologists, and the combination of the two methods, for the identification of patients whose computed tomography (CT) reports raised the concern for lung cancer.

**Methods:**

An NLP algorithm was developed using Clinical Text Analysis and Knowledge Extraction System (cTAKES) with the Yale cTAKES Extensions and trained to differentiate between language indicating benign lesions and lesions concerning for lung cancer. A random sample of 450 chest CT reports performed at Veterans Affairs Connecticut Healthcare System between January 2014 and July 2015 was selected. A reference standard was created by the manual review of reports to determine if the text stated that follow-up was needed for concern for cancer. The NLP algorithm was applied to all reports and compared with case identification using the manual coding by the radiologists.

**Results:**

A total of 450 reports representing 428 patients were analyzed. NLP had higher sensitivity and lower specificity than manual coding (77.3% *v* 51.5% and 72.5% *v* 82.5%, respectively). NLP and manual coding had similar positive predictive values (88.4% *v* 88.9%), and NLP had a higher negative predictive value than manual coding (54% *v* 38.5%). When NLP and manual coding were combined, sensitivity increased to 92.3%, with a decrease in specificity to 62.85%. Combined NLP and manual coding had a positive predictive value of 87.0% and a negative predictive value of 75.2%.

**Conclusion:**

Our NLP algorithm was more sensitive than manual coding of CT chest reports for the identification of patients who required follow-up for suspicion of lung cancer. The combination of NLP and manual coding is a sensitive way to identify patients who need further workup for lung cancer.

## INTRODUCTION

Lung cancer is the most common cause of cancer-related death both in the United States and worldwide.^1,2^ Delays in lung cancer diagnosis and treatment can result from a failure to act upon abnormal radiologic findings in a timely fashion.^3^ To ensure that all patients with imaging findings suspicious for cancer receive appropriate and prompt workup, the Veterans Affairs Connecticut Healthcare System (VACHS) established a cancer care coordination program in 2007.^4^ The program is run by nurse and nurse practitioner teams and uses an interactive database and reminder system: the Cancer Care Tracking System (CCTS). CCTS was developed at VACHS to identify and track patients for whom diagnostic imaging reports raise the possibility of cancer. Imaging reports in which the diagnosis of lung cancer is considered are identified in CCTS both by nationally defined radiology diagnostic codes (cancer alerts) entered by the attending radiologist at the time of image interpretation (manual coding) and by a natural language processing (NLP) algorithm. High-risk radiology studies are reviewed at a weekly tumor board, and all patient cases that require follow-up and/or further workup are tracked using CCTS. The VA radiology coding system is distinct from International Classification of Diseases, Ninth Revision (ICD-9), or other coding systems used for diagnosis or billing. The lung NLP algorithm was developed as an additional safety measure, because internal audits indicated that not all radiology reports of patients with lesions suspicious for cancer were being manually coded as such. The lung NLP algorithm was implemented as part of CCTS at VACHS in February 2011.

The goals of this study were to determine the accuracy and reliability of the diagnostic imaging manual coding process for lung lesions suspected to be cancer outside of the lung cancer screening setting and to compare patient case identification by manual coding with patient case identification using NLP and patient case identification using the combination of both methods on a distinct data set that had not been used for the NLP algorithm development.

## METHODS

### Creation of NLP Coding System

We used the Clinical Text Analysis and Knowledge Extraction System (cTAKES) with the Yale cTAKES Extensions, an open-source, clinical NLP pipeline to process radiology report.^5,6^ The Yale cTAKES Extensions NLP pipeline annotates syntactic structure (eg, sections, sentences, phrases) and semantic content (eg, concepts) and then performs negation detection through a modified NegEx algorithm. We configured cTAKES to map text from radiology reports to concepts from the Unified Medical Language System (UMLS),^7^ a compendium of biomedical vocabularies and ontologies that includes the Systematized Nomenclature of Medicine–Clinical Terms (SNOMED-CT), ICD-9, and others. The UMLS defines semantic relationships between concepts and enumerates synonyms for each concept. We used the semantic relationships in the UMLS to map specific concepts to coarse-grained concepts relevant to the classification of cancer alerts; for example, the terms left upper lobe and lingula would be locations that are mapped to the coarse-grained concept of lung. The classification rules developed can be found in the Data Supplement. The UMLS lacks many concepts specific to the radiology domain (eg, echogenic focus or tree-in-bud opacity). We extended the UMLS and added concepts relevant to the classification of cancer alerts, in particular, concepts that pertain to radiographic abnormalities. In addition, we introduced semantic relationships to define coarse concept groups for this classification task. For example, for the purposes of this classification task, inflammatory and infectious processes were considered synonymous, and we mapped concepts indicative of such processes to a single concept group; for example, the term atelectasis was mapped to the concept group benign. The customized dictionary of additional terms added to SNOMED-CT (2010 version) used for NLP development can be found in the Data Supplement.

**Fig 1.**
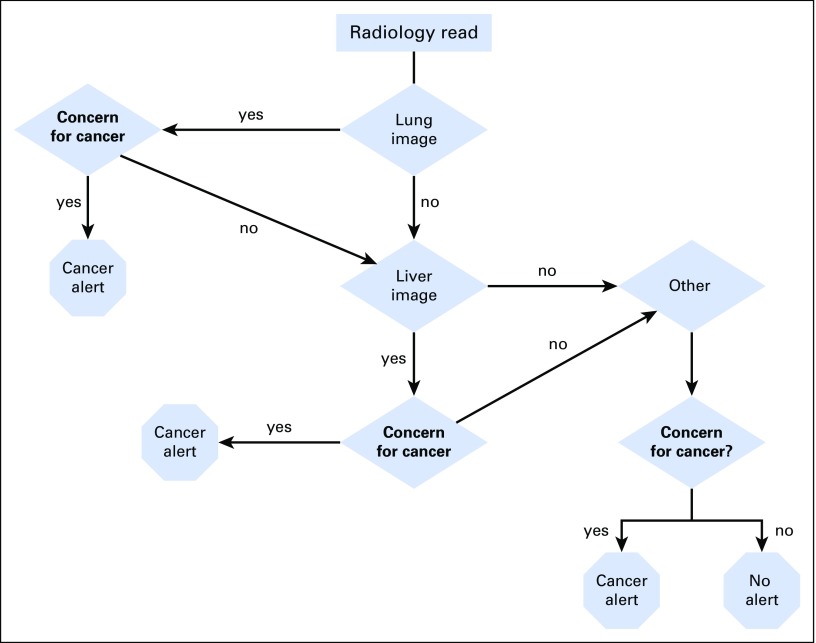
Overall structure of cancer tracker natural language processing algorithm.

**Fig 2.**
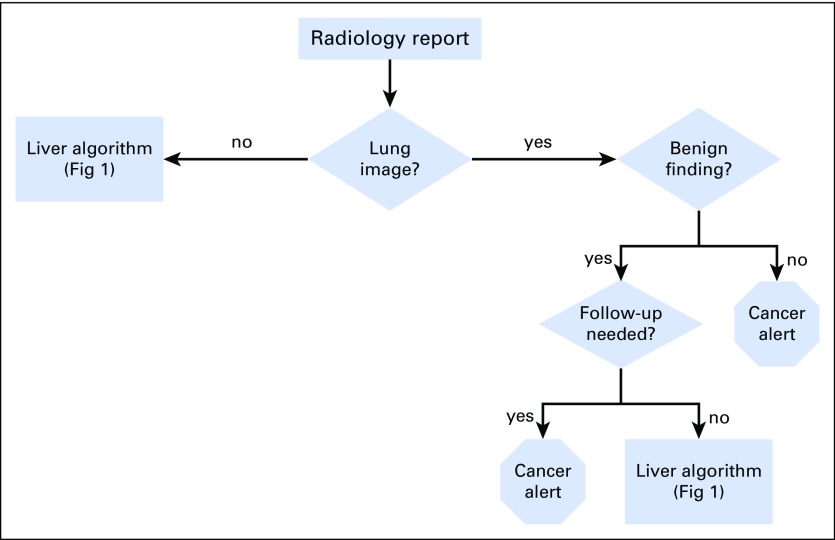
Classification of lung radiology report text.

A team consisting of a radiologist (C.T.), hepatologist (T.T.), and bioinformatician (V.G.) developed rules to extract information from the radiology reports relevant to their classification as cancer alerts. This was based on an initial training corpus of computed tomography (CT) reports that included all chest CT reports done at VACHS from July 2010 through August 2010. We illustrate the overall system in Figure 1 and the lung nodule classification scheme in Figure 2. The system is designed to sequentially evaluate whether there are abnormalities noted in radiology reports concerning for malignancy in the lung, liver, or other structures. The algorithms run sequentially and separately based on the location (lung, liver, or other). If there is an abnormality in any of these areas, a cancer alert is created, and the algorithm moves on to the next report (Fig 1). Figure 2 shows in greater detail how the algorithm categorizes lung lesions as necessitating a cancer alert. The system evaluates each sentence in every radiology report using the following algorithm: It first determines if the sentence describes findings related to the location lung. If yes, it determines if the sentence mentions benign findings; further classification determines whether benign findings will require follow-up, which will trigger an alert. If it is not benign, the system classifies this sentence as a cancer alert. The classification rules can be found in the Data Supplement. CCTS incorporating the NLP algorithm has been running on all CT reports at all VACHS locations since February 2011.

### Sample Selection

This study was approved by our local institutional review board, and the requirement for patient informed consent was waived. On the basis of a nomogram with an anticipated sensitivity of 0.97 and precision of 0.03 and an estimated prevalence of 30% of reads as being positive (ie, suspicious for lung cancer), a sample size of 400 was calculated.^8^ We included in our study a random sample of 450 deidentified chest CT reports performed between January 2014 and July 2015, which were ordered for purposes other than lung cancer screening. The reports included CT chest scans performed with or without contrast, CT angiograms of the chest, and CT scans of chest/abdomen/pelvis or chest/abdomen. Using ICD-9 codes (1 inpatient or 2 outpatient codes), we excluded patients with a known diagnosis of lung cancer in the previous 5 years. The reports included the indication for which the study was ordered as well as the radiologist’s interpretation of the imaging and his or her recommendations. Six reports were excluded because they were duplicate studies.

### Creation of Reference Standard Validation Set

Three of the authors (R.W., M.G.R., A.H.M.) reviewed the radiology reports according to a set of mutually agreed upon rules to determine positive (concern for cancer, requires follow-up) and negative reports. Reviewers were blinded to the manual codes and to results of the NLP. A positive report was any patient case in which the radiologist explicitly stated that follow-up was needed for a lesion concerning for malignancy (both within the lung and elsewhere). Negative reports included those that required follow-up for nonmalignant processes (eg, aneurysms); lymph nodes measuring < 1 cm unless the report explicitly stated that their number, location, or configuration was abnormal; and lung nodules that were < 4 mm and had been stable for at least 1 year. Abstracting rules were initially tested by all three reviewers on 20 reports and were subsequently refined and then applied to an additional 30 reports. The three-reviewer agreement for these 30 reports was calculated as an intraclass correlation coefficient (ICC) of 0.84 (95% CI, 0.73 to 0.92).

### Data Collection

Using the abstracting rules, the remaining 400 reports were each categorized by one reviewer, and ambiguous reports were adjudicated by all three reviewers. For all reports, the manually entered primary and secondary diagnostic codes were identified. Reports with codes that would trigger a cancer alert, such as “64-Lung Nodules for follow-up team” and “73-Possible Malignancy,” were considered positive. The NLP cancer tracker algorithm was run on the same set of radiology reports, and each report was categorized as positive or negative by NLP. For examining combined manual and NLP results, reports were assigned as positive if both or either read was positive.

### Statistical Analysis

True positives and negatives as well as false positives and negatives with manual and NLP coding were determined using the validation set as the reference standard. Sensitivity, specificity, positive predictive value (PPV), and negative predictive value (NPV) were calculated. Subgroup analyses were conducted for studies that were ordered for oncology purposes versus nononcology purposes. SPSS 19.0 software (SPSS, Chicago, IL) was used to calculate ICCs for the 30–patient case interrater reliability study (ICC, mixed model; type, absolute agreement; single measure, alpha = 0.05, test value = 0).

## RESULTS

The median age of the patients was 67.1 years (interquartile range, 62.81-72.93); 2.6% (n = 11) were women, 89% (n = 371) were white, 10.3% (n = 43) were black, and 0.7% (n = 3) were American Indian, Alaskan native, native Hawaiian, or Pacific Islander, and 11 persons were of unknown or missing race. The majority of the imaging was performed in the outpatient setting (93.5%; n = 417). A total of 17 radiologists authored the 446 radiology reports, which included 428 unique patients. Only three radiologists completed fewer than five cases.

Table 1 lists the number of reports read as positive or negative by the reference standard, manual coding, and NLP. Table 2 shows that NLP had a significantly higher sensitivity but significantly lower specificity than manual coding (77.3% *v* 51.5% and 72.5% *v* 82.5%, respectively). NLP and manual coding had similar PPVs (88.4% *v* 88.9%, respectively), but NLP had a significantly higher NPV than manual coding (54% *v* 38.5%, respectively). In Table 3, reports ordered for cancer workup (n = 333) were compared with those ordered for noncancer indications (n = 102). Eleven reports did not indicate the reason for the study and were not included in the subgroup analysis. For both subgroups, sensitivity was higher in the NLP group than in the manual coding group (79.1% *v* 54.9%, respectively, in the cancer-related indication group; 71.1% *v* 37.8%, respectively, in the noncancer indication group). In the noncancer group, NLP read alone had significantly higher sensitivity than manual read alone and borderline significance on higher NPV. Specificity was lower for NLP in both subgroups (60% *v* 73.3%, respectively, in the cancer-related group; 86% *v* 91.2%, respectively, in the noncancer related group).

**Table 1.**
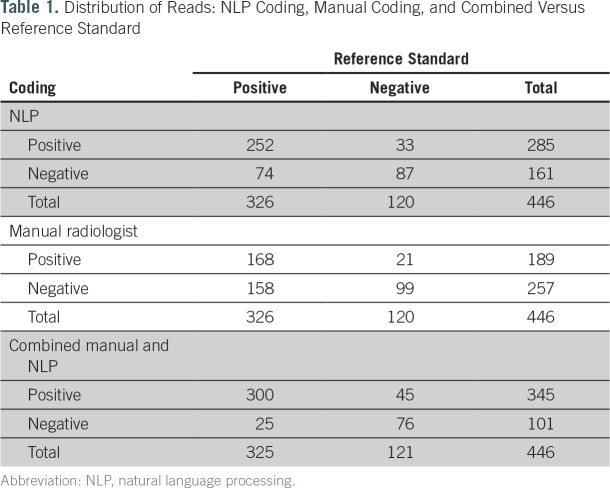
Distribution of Reads: NLP Coding, Manual Coding, and Combined Versus Reference Standard

**Table 2.**
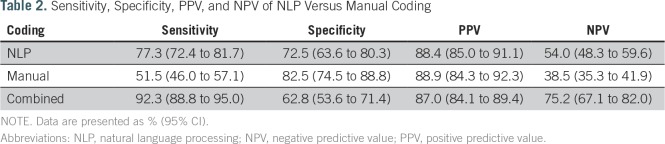
Sensitivity, Specificity, PPV, and NPV of NLP Versus Manual Coding

**Table 3.**
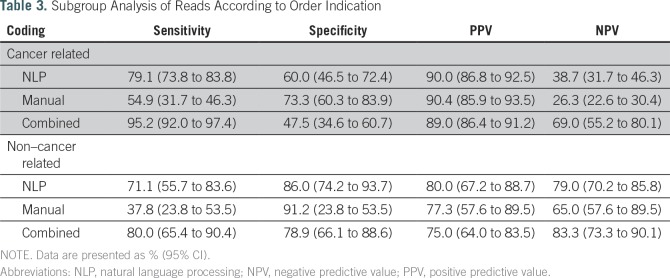
Subgroup Analysis of Reads According to Order Indication

When manual and NLP were combined, we observed an increase in sensitivity (92.3%) and decrease in specificity (62.8%) compared with either method alone (Table 2). The PPV remained the same (87.0%), whereas the NPV increased (75.2%; Table 2). The sensitivity and NPV of combined NLP and manual read were significantly better than those of NLP or manual alone. With respect to performance in cancer-related and non–cancer-related tests for the combined manual and NLP results, sensitivity and PPV were higher in the cancer group, whereas specificity and NPV were higher in the noncancer group (Table 3).

## DISCUSSION

Using manual reviews of lung radiology reports as our reference standard, we found that our NLP was 26% more sensitive than manual coding by radiologists in identifying patient cases that required tracking or further workup for lung cancer (77.3% *v* 51.5%, respectively). The difference in sensitivity between the two methods was especially pronounced in the subgroup of radiology images that were ordered for non–cancer-related indications (71.1% *v* 37.8%). There was a decrease in specificity for both manual and NLP coding when examining the cancer-related indications subset, which is consistent with the literature.^9^ Importantly, when manual coding and NLP were combined, the sensitivity of patient case identification was increased to 92.3%.

Prior studies have been performed examining NLP algorithms to aid in the identification of lung nodules and have shown sensitivity and specificity in the 90% and 70% range, respectively.^10^ However, many past studies used an enriched patient population with electronic health record codes positive for lung nodules. Our system attempts to go a step further and identify not only lung nodules but also lung nodules and other findings that may be suggestive of pulmonary malignancy. Additionally, in contrast with other studies, our study goal was to demonstrate the utility of our system in a non–lung cancer screening population. Prior studies have shown that recommendations for follow-up of imaging of incidental findings may not be acted upon, thus potentially compromising patients’ health.^11^ An automated, reliable method to identify patients with such imaging is a prerequisite for a centralized tracking and coordination system. Our algorithm has been developed to include suspicious findings noted in the lung, liver, or other organs (Fig 1).

Our study is the first to our knowledge to compare NLP and manual coding against a reference standard established by clinicians in unselected radiology reports. NLP applied to radiology reports already selected by ICD codes has been shown to be a sensitive method to identify lung nodules.^12^ In our study, 73% (326 of 446) of the randomly selected chest CT scans had findings that required further workup for malignancy, compared with only 20% to 31% in other populations.^12,13^ This reflects the fact that veterans are at higher risk for lung cancer than the general population, likely because of higher rates of smoking and environmental and combat-related exposures,^14-16^ and further underscores the need for cancer coordination and tracking in this population.

Our group and others have shown that with the increased use of cross-sectional imaging, there is also an increase in the identification of incidentally found malignancies.^17^ Patients with incidental radiology findings are especially at risk for harm related to delays in diagnosis and treatment. At VACHS, 52% of the non–small-cell lung cancers diagnosed between the years of 2005 and 2010 were incidental findings on imaging obtained for other reasons, such as workup of unrelated respiratory symptoms, staging or surveillance of other malignancies, and others.^18^

The main limitations of our study are that it was conducted at a single VA facility. Additional studies are needed to establish the performance of our NLP and cancer care coordination program in other VA centers and health care systems. With the increasing use of electronic medical records, the broad implementation of lung cancer screening, and the increasing use of cross-sectional imaging, the value of automated systems of patient case identification and tracking of lung nodules is likely to increase.
